# Variability of plasma and urine betaine in diabetes mellitus and its relationship to methionine load test responses: an observational study

**DOI:** 10.1186/1475-2840-11-34

**Published:** 2012-07-11

**Authors:** Michael Lever, Sandy Slow, David O McGregor, Warwick J Dellow, Peter M George, Stephen T Chambers

**Affiliations:** 1 Biochemistry Unit, Canterbury Health Laboratories, PO Box 151, Christchurch, 8140, New Zealand; 2 Department of Pathology, University of Otaga Christchurch, Christchurch, New Zealand; 3 Nephrology Department, Christchurch Hospital, Christchurch, New Zealand

**Keywords:** Betaine, Dimethylglycine, Methionine load, Homocysteine

## Abstract

**Background:**

Since betaine is an osmolyte and methyl donor, and abnormal betaine loss is common in diabetes mellitus (>20% patients), we investigated the relationship between betaine and the post-methionine load rise in homocysteine, in diabetes and control subjects. The post-methionine load test is reported to be both an independent vascular risk factor and a measure of betaine sufficiency.

**Methods:**

Patients with type 2 diabetes (*n* = 34) and control subjects (*n* = 17) were recruited. We measured baseline fasting plasma and 4-hour post-methionine load (L-methionine, 0.1 mg/kg body weight) concentrations of homocysteine, betaine, and the betaine metabolite *N,N*-dimethylglycine. Baseline urine excretions of betaine, dimethylglycine and glucose were measured on morning urine samples as the ratio to urine creatinine. Statistical determinants of the post-methionine load increase in homocysteine were identified in multiple linear regression models.

**Results:**

Plasma betaine concentrations and urinary betaine excretions were significantly (*p* < 0.001) more variable in the subjects with diabetes compared with the controls. Dimethylglycine excretion (*p* = 0.00014) and plasma dimethylglycine concentrations (*p* = 0.039) were also more variable. In diabetes, plasma betaine was a significant negative determinant (*p* < 0.001) of the post-methionine load increase in homocysteine. However, it was not conclusive that this was different from the relationship in the controls. In the patients with diabetes, a strong relationship was found between urinary betaine excretion and urinary glucose excretion (but not with plasma glucose).

**Conclusions:**

Both high and low plasma betaine concentrations, and high and low urinary betaine excretions, are more prevalent in diabetes. The availability of betaine affects the response in the methionine load test. The benefits of increasing betaine intake should be investigated.

## Background

Betaine (“glycine betaine”, “trimethylglycine” or “TMG”) is a major osmolyte, accumulated in tissues for cell-volume regulation [1-3], and this tissue store is also a reservoir of methyl groups. Betaine is essential for life, and is obtained either from the diet [4,5] or endogenously by the mitochondrial oxidation of choline [6]. Therefore it is of interest that more than 20% of patients with diabetes mellitus excrete abnormal amounts of betaine in their urine [7,8], and it is worth considering the metabolic consequences of this loss. Betaine catabolism is mediated by betaine-homocysteine methyltransferase which is known to be regulated both by the supply of methyl groups [9] and by osmotic stress [10,11]. Through this enzyme, betaine is a major determinant of circulating homocysteine in mice [12], and in patients with lipid disorders the urinary excretion of betaine is the main factor affecting plasma homocysteine [13,14]. It has been suggested that betaine deficiency is a common feature of the metabolic syndrome [15,16], and may be pathogenic. In a large cross-sectional study, low plasma betaine concentrations were associated with an unfavourable atherogenic risk profile. [17] and subjects with lipid disorders who excreted high levels of betaine appeared to have an increased incidence of vascular disease [18].

The methionine load test has been proposed as a measure of betaine sufficiency [15,16,19], and the rise in homocysteine in this test is an independent risk factor for vascular disease [20,21]. We therefore compared the responses of patients with type 2 diabetes and matched control subjects in the methionine load test.

## Methods

### Methionine load test study

The study was approved by the Canterbury Ethics Committee (New Zealand) and all subjects gave written informed consent. Patients with Type 2 diabetes were identified through the Canterbury retinal screening programme database and invited to participate. Controls were recruited by posting notices around the hospital campus. Thirty four patients (aged 51–75 yrs, 20 males) and 17 controls (aged 54–69 yrs, 10 male) were recruited. They were asked to fast for 16 hours and to withhold morning medications. Their height, weight, age, and medical history were recorded. Venous blood was drawn into EDTA tubes that were placed on ice immediately for determining fasting plasma betaine and homocysteine concentrations and a urine sample was collected. They were then given a methionine load of 0.1 g/kg body weight of L-methionine dissolved in 100 ml fruit juice. They also ate a standard snack consisting of a jam (jelly) sandwich to avoid hypoglycaemia. They took no further food or fluid until their return 4 hours later, when a second venous blood sample was collected. The betaine, dimethylglycine and homocysteine from the pre- and post- methionine load blood samples were measured in the same analytical batches.

### Analytical methods

Betaine and *N,N*-dimethylglycine were measured in plasma and urine by high performance liquid chromatography (HPLC) as their 2-naphthacyl derivatives [22,23]. Plasma homocysteine was measured by fluorescence polarization on an Abbott IM_X_ Analyzer (Abbott Laboratories). Creatinine was measured in plasma and urine using the Jaffé reaction on the fully automated Abbott Aeroset Analyzer (Abbott Laboratories, USA). Serum vitamin B_12_ and red blood cell (RBC) folate concentrations were measured by separate competitive immunoassays on an automated Chemiluminescence ACS:180 Analyzer (Chiron Diagnostics Corporation; USA). Hemoglobin A_1c_ was measured by the Diamat fully automated glycosylated hemoglobin analyzer system (Bio-Rad) and were expressed as a percentage of hemoglobin.

### Statistical methods

Urine betaine and *N,N*-dimethylglycine excretions were positively skewed, and in the subjects with diabetes plasma betaine, dimethylglycine, homocysteine and serum vitamin B_12_ concentrations were also positively skewed. Where the raw data was not normally distributed (Kolmogorov-Smirnov test) the data were log transformed prior to conducting correlation and regression analyses. Associations were evaluated as Pearson’s correlation coefficient and from multiple linear regression models. The regression models were based on the most significant variables that consistently appeared in at least 12 best subsets regression models (not reported), and only significant variables are included in the final models presented. Statistical analyses and figure preparation were carried out using SigmaPlot for Windows version 11.2 (Systat Software Inc).

## Results

### Characteristics of the groups

The subjects with diabetes were slightly older than the control subjects (Table 1). Almost all subjects were folate and vitamin B_12_ replete. The fasting blood glucose concentrations of the subjects with diabetes were, as expected, higher than in the controls (Table 1), and the hemoglobin A_1c_ data shows that the subjects with diabetes had varied degrees of control.

**Table 1:** Characteristics of subjects in methionine load test study

	*N*	Median	Full range
*Females with diabetes:*			
Age (y)	14	67.5	60 – 74
Body Mass Index	14	27.0	17.1 - 37.6
Fasting plasma glucose (μmol/L)	14	7.4	5.5 - 10.2
Serum vitamin B_12_ (pmol/L)	14	337	215 – 587
Red cell folate (nmol/L)	14	677	508 – 964
Hemoglobin A_1c_ (%)	14	7.75	6.7 - 9.8
*Males with diabetes:*			
Age (y)	20	69	51 – 75
Body Mass Index	20	27.2	22.1 - 42.3
Fasting plasma glucose (μmol/L)	20	7.85	4.8 - 14.8
Serum vitamin B_12_ (pmol/L)	20	330.5	129 – 697
Red cell folate (nmol/L)	20	655.5	406 – 1046
Hemoglobin A_1c_ (%)	20	7.4	5.3 - 9.0
*Female control subjects:*			
Age (y)	7	63	55 – 69
Body Mass Index	7	28.7	24.9 - 33.5
Fasting plasma glucose (μmol/L)	7	4.5	3.9 - 5.8
Serum vitamin B_12_ (pmol/L)	7	357	302 – 580
Red cell folate (nmol/L)	7	840	546 – 1071
*Male control subjects:*			
Age (y)	10	57	54 – 66
Body Mass Index	10	26.8	21.1 - 36.5
Fasting plasma glucose (μmol/L)	10	4.75	3.9 - 5.8
Serum vitamin B_12_ (pmol/L)	10	355.5	227 – 837
Red cell folate (nmol/L)	10	847	507 – 971

### Differences between subjects with and without diabetes

**Figure 1:**
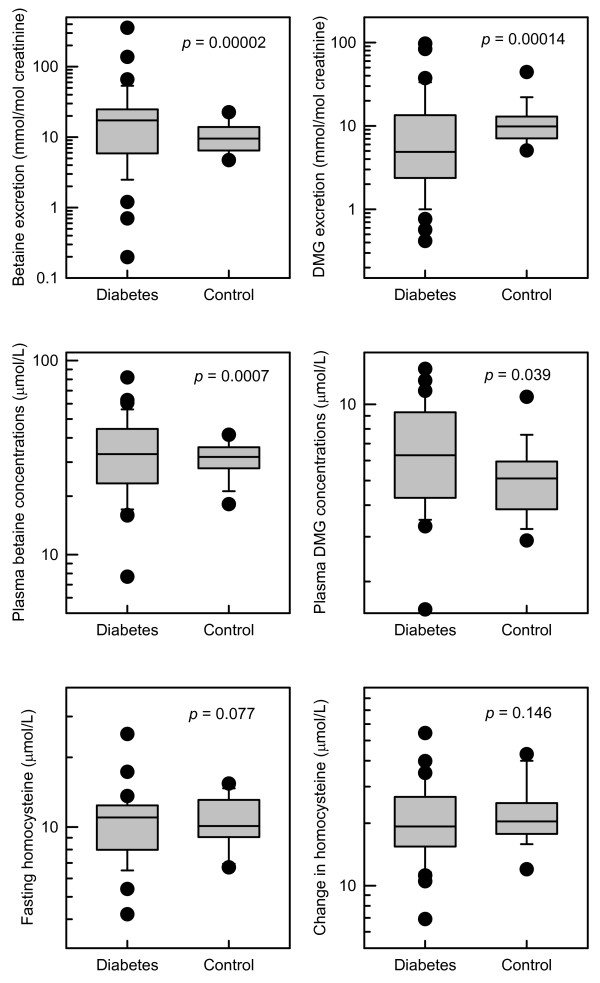
**Variability of betaine and homocysteine metabolites.** Variablity in subjects with diabetes (n = 34) and control subjects (n = 17). DMG: *N,N*-dimethylglycine. The *p* values are the significance of the differences between the variances of the subjects with and without diabetes, calculated using log-transformed data. The change in homocysteine is the difference between the plasma homocysteine before and 4 h after a methionine load; plasma betaine and homocysteine concentrations are fasting, before a methionine load.

In this study, the medians of fasting plasma betaine and *N,N*-dimethylglycine concentrations, fasting plasma total homocysteine, the rise in homocysteine, and the excretions of betaine and *N,N*-dimethylglycine, were not significantly different between subjects with diabetes and controls (Figure 1). The striking difference is the greater variability of plasma betaine concentrations, and urine betaine excretions (Figure 1), in the subjects with diabetes. A similarly increased variability is apparent with *N,N*-dimethylglycine. The difference in variability was less pronounced for fasting plasma homocysteine and the rise in homocysteine after a methionine load (Figure 1), and a trend for higher variability in the subjects with diabetes was not significant in this study.

The post-methionine load plasma betaine and homocysteine concentrations were also significantly more variable in the subjects with diabetes (on log-transformed data, *p* < 0.001 for betaine and *p* = 0.0016 for *N,N*-dimethylglycine), as was the post-methionine change in plasma *N,N*-dimethylglycine (*p* = 0.013), but not the post-methionine change in plasma betaine (*p* = 0.7).

### Predictors of the post-methionine load increase in homocysteine

**Figure 2:**
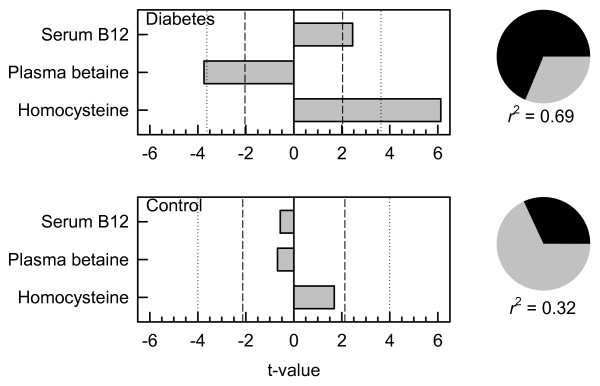
**Multiple linear regression model for the post-methionine load increase in homocysteine.** Top, in subjects with diabetes, bottom, control subjects. Dependent variable: log(rise in plasma homocysteine). Independent variables: log(serum vitamin B_12_), log(post-methionine plasma betaine) and log(baseline fasting homocysteine). Bars show the significance of the factors as t-values, negative for a negative relationship and positive for a positive relationship; dashed lines *p* = 0.05; dotted lines *p* = 0.001. Pie graphs at the right show the proportion of variance (dark segment) explained by regression: unexplained variance in grey.

In the subjects with diabetes, the statistically significant (*p* < 0.05) predictors of the post-methionine load increase in plasma homocysteine, identified by best subset regression, were fasting plasma homocysteine, either fasting or post-methionine load betaine, and (in most models) serum vitamin B_12_. Variables that did not enter any of 36 models with *p* < 0.1 included age, body mass index, red blood cell folate, hemoglobin A_1c_, plasma glucose concentrations, plasma *N,N*-dimethylglycine concentration (either fasting or post-methionine load), and the urinary excretions of glucose, betaine and *N,N*-dimethylglycine. Log-transformed variables were used where the raw data was not normally distributed (Kolmogorov-Smirnov test). The three strongest candidate predictors (all log-transformed) were included in an explicit multiple linear regression model (Figure 2) which confirmed that the fasting plasma homocysteine was the strongest (positive) predictor, and the post-methionine load plasma betaine concentration was also a highly significant (*p* < 0.001) negative predictor. The serum vitamin B_12_ concentration also a positive predictor (*p* = 0.021). The model explained most of the variance in the post-methionine load increase in homocysteine (*r*^2^ = 0.69). When the fasting plasma betaine concentration was included in the model instead of the post-methionine concentration, a similar result was obtained (*r*^2^ = 0.56) but the fasting betaine concentration appears to be a weaker negative predictor (*p* = 0.013).

The best subsets models of the post-methionine load increase in plasma homocysteine calculated on the control subjects either showed no significant predictors, or the fasting plasma homocysteine concentration appeared as a sole significant predictor. The same three-factor explicit multiple regression model was generated using the control data (Figure 2); this was a poorer model than when the subjects with diabetes were used (*r*^2^ = 0.32) and none of the terms were significant.

Regression models were also estimated using all subject data (combined controls and subjects with diabetes). In the three variable linear regression model (*r*^2^ = 0.58) both fasting homocysteine (positive) and post-methionine load plasma betaine (negative) were significant predictors (*p* < 0.001) but vitamin B_12_ was not significant (p = 0.077). If diabetes (0 or 1) was included as a variable in best-subsets regression models it was in no case significant. Age also did not enter any of the models as a predictor.

### Glucose, betaine and homocysteine

Betaine excretion positively correlated with glucose excretion (log transformed data; *r* = +0.47, *p* = 0.005, *n* = 34) in patients with diabetes. Dimethylglycine excretion also correlated with glucose excretion (log transformed data; *r* = +0.53, *p* = 0.001, *n* = 34). There was an inconclusive correlation between plasma glucose and log-transformed betaine excretion (*r* = +0.33, *p* = 0.056, *n* = 34); the Spearman’s correlation coefficient was significant (*r*_*s*_ = +0.37, *p* = 0.03). Neither fasting homocysteine, nor the post-methionine load increase in homocysteine, correlated with either plasma glucose or glucose excretion. Hemoglobin A_1c_ did not strongly correlate with betaine excretion, but there was a significant positive correlation (*r* = +0.52, *p* = 0.003, n = 30) between hemoglobin A_1c_ and the post-methionine load change in plasma dimethylglycine (measured as the difference in log(plasma dimethylglycine) before and after the load). Hemoglobin A_1c_ did not correlate with either fasting homocysteine or the post-methionine load increase in homocysteine.

### Differences between subjects with high and low betaine excretions

**Figure 3:**
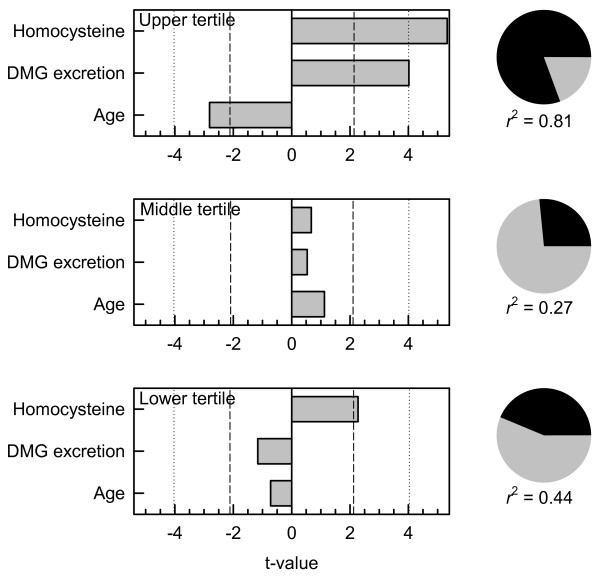
**Multiple linear regression model for the post-methionine load increase in homocysteine.** Data on subjects with diabetes and controls combined. Top, subjects in the highest tertile of betaine excretion and bottom in the lowest tertile of betaine excretion. Dependent variable: log(rise in plasma homocysteine). Independent variables: log(baseline fasting homocysteine), log(dimethylglycine excretion as mmol dimethylglycine per mole creatinine) and age (years). Bars show the significance of the factors as t-values, negative for a negative relationship and positive for a positive relationship; dashed lines *p* = 0.05; dotted lines *p* = 0.001. Pie graphs at the right show the proportion of variance (dark segment) explained by regression: unexplained variance in grey.

Data from all 51 subjects were combined and ranked on betaine excretion. The factors affecting the post-methionine load increase in homocysteine were identified in the highest and lowest tertiles of betaine excretion (n = 17 in each group) using best-subsets regression. In the highest betaine excretion group the three significant predictors of the increase in plasma homocysteine (expressed as log(rise in homocysteine)) were fasting homocysteine (log transformed), dimethylglycine excretion (log transformed) and age, and in an explicit three-variable multiple linear regression model (Figure 3) these explained 81% of the variance of the rise in homocysteine. Fasting homocysteine and dimethylglycine excretion were significant (p < 0.001) positive predictors whereas age was a negative predictor. This three variable model was a poorer predictor in the middle and lower tertile groups of betaine excretion (Figure 3), explaining <50% of the variance, and in the lower tertile there was a non-significant trend for dimethylglycine excretion to be negatively associated with the rise in homocysteine.

The differences between tertiles were also detected by Pearson correlation coefficients between the rise in homocysteine and fasting homocysteine (both log transformed) and with dimethylglycine excretion (also log transformed). In the upper tertile these were *r* = +0.70 (*p* = 0.0018) and +0.61 (*p* = 0.009) respectively; in the lower tertile they were *r* = +0.61 (*p* = 0.010) and −0.41 (*p* = 0.10). In the case of dimethylglycine excretion the two correlation coefficients were significantly different (*p* = 0.0024). This was further evaluated by comparing the highest and lowest quartiles of betaine excretion; in the highest quartile (n = 13) the corresponding correlation between the rise in homocysteine and dimethylglycine excretion was *r* = +0.61 (*p* = 0.027) and in the lowest quartile it was *r* = −0.62 (*p* = 0.024); despite the small numbers these are significantly different (*p* = 0.0013). In both quartiles the fasting homocysteine was a positive predictor of the post-methionine rise in homocysteine, *r* = +0.86, *p* = 0.00002 in the highest quartile and *r* = +0.65, *p* = 0.017 in the lowest quartile.

## Discussion

Betaine is accumulated by most tissues as an osmolyte, with tissue concentrations much higher than plasma concentrations [3]. This tissue betaine is a major store of methyl groups. Mobilizing betaine involves methylating homocysteine to methionine, with the production of *N,N*-dimethylglycine [9], and betaine is probably the main regulator of non-fasting plasma homocysteine [9,12,24]. High urinary betaine loss is common in diabetes mellitus [7,8,25], and may persist for years [26], potentially causing a betaine insufficiency that could be expected to have complex consequences, affecting both the ability of cells to maintain their volume and the supply of methyl groups essential for normal metabolism [15,16,27]. Other causes of deficiency could include a dietary insufficiency of choline plus betaine. Betaine is either obtained from the diet or by the mitochondrial oxidation of choline [15,28], and defective conversion of choline to betaine is another possible cause.

The most obvious difference between the subjects with diabetes and the control subjects is the greater variability in plasma and (especially) urine betaine in the patients with diabetes. This substantiates the impression presented visually in an earlier report [29], and could be explained by a higher prevalence of both elevated and decreased plasma and urine betaine in subjects with diabetes compared with the healthy population. Metabolites related to betaine, such as dimethylglycine and homocysteine, may also be more variable in diabetes, but a larger study would be needed to confirm this. Superficially, it appears that in the subjects with diabetes, plasma betaine is a stronger predictor of the post-methionine load rise in homocysteine than it is in the control subjects; however, this could be an artefact of the greater variance (in diabetes) of all the factors in the regression model. This possibility is supported by the failure of diabetes to appear as a significant factor when regression models are generated using the population of all subjects. Thus we confirm the findings of Holm et al. [19] about the importance of betaine in determining the post-methionine rise in homocysteine. Our finding that folate status failed to enter any of the regression models is also in agreement with Holm et al. [19]. Despite the possibility of bias being introduced by the age difference between subjects with diabetes and the control subjects, age was not a significant predictor of the post-methionine rise in homocysteine in any model, even those based on the combined population of all subjects.

This observational study cannot explain high and low plasma betaine concentrations in diabetes. Plasma betaine concentrations are normally tightly controlled [15,25] and lower than intracellular concentrations [3]; efflux from cells is both osmoregulated and hormonally controlled. Compromise of this control could lead to elevated plasma concentrations. Low plasma concentrations may indicate a deficiency, for example as a result of urinary loss, defective synthesis from choline or poor dietary choices. High urinary excretion is presumably a renal phenomenon [15] and may be associated with the role of betaine as a renal osmolyte; low excretion is more likely to reflect a genuine deficiency. Methyl metabolism, including betaine metabolism, is disturbed in a rat model of diabetes [27] and suggests other mechanistic models to investigate: plausibly, the dual role of betaine could connect disturbed methyl metabolism with disturbed osmoregulation.

The post-methionine load increase in homocysteine has been proposed as a test for betaine insufficiency [15-19]. This study supports this, though it was too small to answer the question of whether elevated post-methionine load responses were more common in diabetes, which would imply a greater prevalence of betaine insufficiency in diabetes. The subjects with diabetes and controls had similar ranges of BMI and there were fewer subjects with high betaine excretion in the diabetes group than we have found in other studies. Thus the negative result is inconclusive. However, the effect of betaine on this test was confirmed. The load test is commonly regarded as an independent risk factor for vascular disease [20,21] though its value has been questioned [30]. Betaine supplements have been shown to moderate the post-methionine load increase in homocysteine in normal subjects [19,31] and in renal failure patients [32]. The relationship between dimethylglycine excretion and the post-methionine load homocysteine response suggests hypotheses that could be tested. Dimethylglycine is the by-product of betaine-mediated homocysteine metabolism and an increased production of it implies an increased remethylation of homocysteine; the negative correlation between the two in some subjects with low betaine excretion suggests that this subgroup at least has a primary betaine insufficiency (insufficient betaine to moderate the rise in homocysteine), possibly because of an inadequate intake of choline plus betaine, or a defect in the mitochondrial oxidation of choline to betaine. The low correlations in the group of subjects around the median betaine excretion are likely to reflect the lower variances (in that group) of the variables.

This study does not address mechanisms for the elevated loss of betaine in many subjects with diabetes. However, betaine loss in at least some subjects with diabetes is associated with poor glucose control, and the correlation with urine glucose (rather than with plasma glucose) is consistent with this being a renal effect. It could be informative to investigate whether an increased production of sorbitol (another renal osmolyte) from glucose is a contributing mechanism, since sorbitol excretion is also elevated in diabetes and correlates with betaine excretion [7]. However, this model would need to be reconciled with the observation in a sheep model that elevated glucose itself does not directly cause an increased excretion of betaine [33].

Our results suggest that the supply of betaine may sometimes be limiting. Low plasma betaine has been associated with an unfavourable risk profile in observational studies [14,32], but conversely (in another observational study) high plasma betaine has been associated with an increased risk of vascular events [34]. Since betaine, a major osmolyte, is well-known to be a protein-stabilizing compatible solute [35] and its intracellular concentrations are orders of magnitude higher than those in circulation [3] it is unlikely that an increase in circulating betaine can cause damage, though it may indicate a pathological process, as is seen in animal models when betaine-homocysteine methyltransferase is absent or inhibited [24,36]. Another possible pathological process is a failure of the tight control of betaine efflux from cells. Thus it is plausible that both high and low plasma betaine concentrations may reflect pathological processes. However, betaine supplementation can increase plasma betaine, lower plasma homocysteine, and is safe [37-39]. Betaine intake can be also be increased by dietary choices [4,5]. We suggest that urinary betaine excretion should be measured in patients with diabetes to identify those with excessive betaine loss. Such patients should be encouraged to increase their betaine intakes with high betaine foods such as whole wheat products [40,41], spinach and beets [4,5] or with betaine supplements.

## Conclusions

In diabetes high and low plasma betaine concentrations are more common than in healthy subjects. Both high and low urinary betaine excretions are also more prevalent in diabetes, with the high excretions being associated with glucosuria. The availability of betaine affects the response in the methionine load test but it is not conclusively shown that this effect is more pronounced in diabetes. It would be interesting to investigate whether betaine supplementation of subjects with diabetes and abnormal betaine markers alters their risk of developing vascular disease.

## Competing interests

There are no competing interests to declare.

## Authors’ contributions

ML, SS analyzed the data and took the main responsibility for preparing the MS. ML is responsible for the laboratory in which assays were carried out. DOM had the primary responsibility for the initial design, for recruiting subjects and supervising the methionine load test. WJD assisted DOM in recruitment and conducting the study, carried out the betaine assays in the laboratory and made an initial attempt at analyzing and interpreting the results.PMG had overall supervision and clinical input and input into interpreting the results and edited the MS during preparation. STC was partly responsible for the initial design and has overseen the work at all stages and edited the MS. All authors approve this submission.
